# Current News

**Published:** 1900-03

**Authors:** 


### Current News.

### INTERNATIONAL DENTAL CONGRESS.

The Sub-committee on Transportation has completed arrangements with the well-known tourist firm of Thomas Cook & Sons, 251 Broadway, New York, so that dentists who expect to attend the Congress to be held in Paris, commencing August 8, 1900, may secure for themselves and family steamship and railroad tickets and hotel accommodations at the minimum of expense and trouble.

In making these arrangements the committee have taken into consideration that while some of the delegates may wish to secure only transportation from New York to Paris and back to New York, many delegates will wish to visit other parts of Europe during the summer, and they have planned the following tours to assist such in the selection of a trip that the time at their disposal and their means will suggest.

TOUR 1.

A. From New York by Red Star Line steamer "Friesland" on July 18th for Antwerp, thence rail *via* Brussels to Paris, returning same way to New York. First class passage, providing berth at minimum rate for two-berthed room, $157.85.

If travelling second class from Antwerp to Paris and return, fare would be $4.65 less.

By travelling on steamers "Kensington" or "Southwark" of the same line, fare would be reduced.

B. *Via* Cherburg (North German Lloyd service).

From New York by North German Lloyd steamers "Barbarosa" and "Friederich der Grosse," sailing July 12th and 19th, respectively, for Cherburg, thence rail to Paris and return same way (twin screw service only). First class passage, providing berth in room for two persons (minimum rate), $177.00

C. *Via* Cherburg (Hamburg American Line Service).

From New York by Hamburg American Line steamers "Pennsylvania" and "Pretoria," sailing July 14th and 21st, respectively, to Cherburg, rail to Paris and return *via* Boulogne-sur-mer and Hamburg American steamer (twin screw service) to New York. First class passage, providing minimum fare for berth in room for two persons only, $184.25.

Lower fares can be obtained if occupying berth in room with two or three occupants.

D. *Via* Boulogne-sur-mer (Holland American Line).

From New York by twin screw steamers "Potsdam," "Statendam," and "Rotterdam," sailing July 7, 14, and 28, respectively, to Boulogne-sur-mer, thence rail to Paris and return same way to New York. First class passage, providing minimum fare for berth in room for two passengers, $163.00

If traveling second class from Boulogne to Paris and return, fare would be $3.80 less.

Lower fares can be made by leaving on steamer "Sparndam" July 19th.

Tickets can also be arranged *via* Southampton or Liverpool at proportionate fares.

TOUR 2.

To provide hotel accommodation in Paris for two weeks (fourteen days and thirteen nights) at Grand Hotel du Trocadero, carriage drives for three days, including excursion to St. Cloud and Versailles, twenty tickets of admission to Exposition, and transfers to and from railway station to hotel, $65.00

TOUR 3.

One week's tour to Switzerland from Paris, visiting Lucerne, Interlaken, Thun, Berne, Lausanne, Lake Leman, Geneva, including hotel accommodation, sight-seeing, etc., second class railroad, $50.00.

TOUR 4.

One week's tour from Paris to Mayence, thence steamer on Rhine to Cologne, rail to Amsterdam, The Hague, Rotterdam, Antwerp, Brussels, Harwich, London, including second class railway travel, first class on steamers, hotel coupons (three meals per day with lodging), $42.50.

Those travelling *via* Cherburg can return by steamers of same line from Southampton, and so make a short tour from Continent through England in connection.

There is a United States revenue tax of five dollars upon each ticket, regardless of the number of passengers in whose name it may be made out.

Should anyone wish to make a longer tour than any of the foregoing, or one with a different route, Messrs. Cook & Sons have such a large variety of tours already planned that there need be no difficulty in making a selection to suit the taste, means, or the time at the disposal of anyone.

The war in South Africa has caused the withdrawal of many of the English steamships. Passenger accommodations across the Atlantic will be less than usual this summer, while the Paris Exposition is attracting great numbers, so that the committee wish to impress upon delegates the great importance of securing their steamship accommodations at once.

Address all communications regarding steamships, railroads, hotels, etc., to Messrs. Thomas Cook & Sons, 251 Broadway, New York.

A. W. HARLAN, W. W. WALKER,
W. E. GRISWOLD, WILLIAM JARVIE, Chairman,
*Transportation Committee.*

---

### BOARD OF DENTAL EXAMINERS, COMMONWEALTH OF PENNSYLVANIA.

THE Board of Dental Examiners of the State of Pennsylvania will conduct examinations simultaneously in Philadelphia and Pittsburg, May 8th, 9th, and 10th, and in Philadelphia, June 19th, 20th, and 21st.

Application for examination must be made to Hon. James W. Latta, Secretary of the Dental Council, Harrisburg, Penna.

G. W. KLUMP,
*Secretary.*

WILLIAMSPORT, PENNA.

### MASSACHUSETTS BOARD OF REGISTRATION IN DENTISTRY.

A MEETING of the Massachusetts Board of Registration in Dentistry, for the examination of candidates, will be held at 563 Tremont Street, Boston, Wednesday, March 21, 1900, at 9.30 A.M. Examination in Operative Dentistry at 10 o'clock.

Each candidate must come prepared with rubber-dam, gold, and instruments to demonstrate his skill in operative dentistry. Any-one who wishes may bring his patient. So far as possible patients will be furnished.

The theoretic examination will include operative dentistry, prosthetic dentistry, crown- and bridge-work, orthodontia, anatomy, histology, surgery, pathology, materia medica, therapeutics, physiology, anæsthesia, chemistry, and metallurgy, and will be held at Civil Service Rooms, State House, commencing Thursday, March 22, at 9.30 A.M.

All applications, together with the fee of twenty dollars, must be filed with the secretary of the Board on or before March 14th, as no application for this meeting will be received after that date.

*Candidates who have taken an examination,* and desire to come before the Board again at this meeting, must notify the secretary as above in order to be registered.

G. E. MITCHELL, D.D.S.,
*Secretary.*

25 MERRIMACK STREET, HAVERHILL, MASS.

---

### MISSOURI STATE BOARD OF DENTAL EXAMINERS.

OWING to the fact that Section 2, of New Rule 8, adopted by the National Association for Dental Faculties at Niagara Falls, fixes the requirements of entrance into all associated colleges, the same as required by the State Board of Dental Examiners for the State of Missouri, I am frequently asked for an exact copy of the rules of the Missouri Board for conducting preliminary examinations.

To comply with a request so general, you will kindly publish our rule in full on that point. It is as follows:

### Article II.

Section I.—All colleges operating in this State must comply with the rules as stated in Article I, of the "Rules for Colleges." Also with the following demands of this Board, before their graduates will be granted a Board certificate :

(1) Students applying for admission to a dental college in this State, who cannot present a certificate as required by Rule 8, of Article I, shall then submit themselves to an examination as indicated in Rule 2, Article I.[1]

(2) These examinations must be conducted by a person appointed by the State Superintendent of Public Instruction, through the Secretary of the Board.

(3) It shall be the duty of the person conducting these examinations to make a verified report to the Dean of the College, giving applicant's full name and average grade made by each applicant, and to the Secretary of each Board a duplicate report with each applicant's examination papers, which shall be kept by the Secretary for a period of four years, subject to inspection at any time.

(4) The average grade of an applicant shall be seventy per cent. before he can be admitted to any dental college in the State of Missouri.

(5) It shall be the duty of the Secretary of this Board to notify the Dean of the colleges, whose applicants have been examined, of those who have made the required grade, and are entitled to enter the college as students in the Freshmen year class.

(6) The Deans of colleges admitting students into the Junior or Senior year classes, must in each case make a verified statement to the Secretary in regard to the qualifications upon which each student was admitted.

(7) All expenses incurred in examination of students must be paid by their respective colleges.

(8) It shall also be the duty of this Board to elect two of its members each year to inspect the dental colleges in this State, and they must make a written report at the May meeting of the Board as to their equipment and teaching qualification.

Any further information as to the details of the examination will be furnished upon application.

S. C. A. Ruby,
*Secretary State Board of Missouri.*

---

[1] Rule 2, Article I, sets out the standard of admission,—certificate of entrance into second year high school, etc.

